# Development and validation of machine learning models to predict prediabetes using dietary intake data in young adults in Korea: a cross-sectional study

**DOI:** 10.7586/jkbns.24.029

**Published:** 2024-11-25

**Authors:** Myoung-Lyun Heo

**Affiliations:** Department of Nursing, Jeonju University, Jeonju, Korea

**Keywords:** Prediabetic state, Machine learning, Diet surveys, Young adult

## Abstract

**Purpose:**

This study aimed to develop and compare machine learning models for predicting prediabetes in young adults in Korea using dietary intake data and to identify the most effective model.

**Methods:**

Data from the ninth Korea National Health and Nutrition Examination Survey were used, with 823 participants aged 19–35 years selected after excluding those with missing data. Logistic regression, k-nearest neighbors, and random forest models were applied to predict prediabetes, and the analysis was conducted using the Orange 3.5 program. Five-fold cross-validation was performed to reduce performance variability, and test data were used for final model validation.

**Results:**

In the dataset, 14%–15% of participants were classified as having prediabetes. The random forest model showed the highest performance in terms of classification accuracy, harmonic mean of precision and recall, and precision. Logistic regression had the highest performance regarding the model’s ability to distinguish between individuals with and without prediabetes. Age, thiamine intake, and water intake emerged as the most important predictors.

**Conclusion:**

This study demonstrated the utility of using dietary intake data to predict prediabetes in young adults. The random forest model provided the highest prediction accuracy, supporting early detection and intervention, which could help to reduce unnecessary treatment. This highlights nurses’ important role in educating patients about lifestyle changes and implementing preventive care. Future studies should incorporate additional factors, such as psychological and lifestyle variables, to improve the model's performance.

## INTRODUCTION

Diabetes mellitus is a metabolic disorder characterized by chronically elevated blood glucose levels due to insufficient insulin secretion or insulin resistance. According to the World Health Organization, inadequate long-term blood glucose control can lead to severe complications, affecting the nervous system, kidneys, and cardiovascular system [1]. In South Korea, diabetes is considered a significant public health issue. While the incidence of diabetes among individuals aged 40 years and older has decreased, with a drop of approximately 0.1% annually from 2006 to 2015, national cohort studies show a concerning rise in diabetes prevalence among young adults aged 20–39 years. Specifically, the rate increased from 0.5 to 0.7 per 1,000 individuals in the 20–29 age group and from 2.0 to 2.6 per 1,000 in the 30–39 age group, highlighting the need for increased societal attention [2].

The prevalence of prediabetes, a condition where blood sugar levels are higher than normal but not yet high enough to be classified as diabetes, has also been rapidly increasing among young adults in South Korea [3]. Individuals with prediabetes are at high risk of the condition progressing to diabetes. Early intervention, including lifestyle modifications, during this stage, can effectively prevent the onset of diabetes. Therefore, accurately predicting prediabetes in individuals is crucial for public health strategies aimed at diabetes prevention [4].

Diabetes is one of the chronic diseases caused by lifestyle factors, and the primary reason for the increased risk of diabetes in young adults can be attributed to lifestyle changes that lead to obesity [5]. During the coronavirus disease 2019 pandemic, social distancing and the increase in remote work resulted in decreased physical activity, accompanied by mental health issues such as depression and anxiety. These factors contributed to unhealthy eating habits and the proliferation of irregular diets, which in turn increased the rate of obesity [6,7]. Therefore, assessing nutritional status can provide valuable information for predicting diabetes or prediabetes.

However, accurately assessing dietary intake is a challenging task. Therefore, methodologies such as the 24-hour dietary recall are useful for comprehensively evaluating nutrient intake [8]. The 24-hour dietary recall requires participants to meticulously record all foods and beverages consumed in the past 24 hours, making it a reliable tool for precisely assessing habitual dietary patterns and nutrient intake [9]. The method enables the collection and analysis of important data related to an individual's diet, including carbohydrates, sugars, fiber, and protein, to inform preventive interventions for diabetes [10]. Therefore, dietary intake data obtained through 24-hour dietary recalls can provide valuable information for predicting individuals with prediabetes.

Previous studies have primarily focused on causal analyses and meta-analyses that explore the relationship between specific nutrients and diabetes [11]. While these approaches are advantageous for evaluating the interactions between single variables, they may yield lower prediction accuracy in complex data environments. By contrast, prediction modeling using machine learning accounts for the interactions among multiple variables and employs algorithms capable of efficiently addressing non-linearity and high dimensionality. This enables machine learning models to maintain high predictive accuracy even with complex datasets, capturing intricate patterns that might be overlooked in causal analysis to support better decision-making [12]. Since diabetes can be influenced by a combination of factors beyond nutrition, it is essential to use machine learning models that can integrate and analyze these various variables.

Therefore, integrating various dietary intake data with individual characteristics and applying machine learning algorithms can enable high-accuracy predictions for identifying individuals with prediabetes. This study aims to verify a model for the early prediction of prediabetes in young adults by utilizing dietary intake data, which is easier to collect than blood tests. This model will provide a basis for early interventions for improving the health management of young adults.

### Purpose

This study aims to verify the effectiveness of machine learning models for predicting individuals with prediabetes using dietary intake data from young adults in Korea and to propose the most effective prediction model.

## METHODS

### 1. Study design

This study is a secondary data analysis using the first-year data of the 9th Korea National Health and Nutrition Examination Survey (KNHANES), which was released by the Korea Disease Control and Prevention Agency (KDCA) in January 2024.

### 2. Samples

#### 1) KNHANES

The KNHANES was conducted with the approval of the Institutional Review Board of the KDCA (2018-01-03-4C-A), and the researcher obtained permission from the KDCA to download and analyze the anonymized data for research purposes. For the analysis, this study utilized data from the health and nutrition surveys and medical examinations and integrated data from the various surveys within the same cycle. After aligning IDs, only data without missing values were selected for analysis. Although the KNHANES data were collected using a complex sampling design, which recommends applying complex sample analysis, this study primarily aims to predict and learn data patterns using machine learning rather than estimating specific population statistics. Therefore, sampling weights were not applied [13].

### 3. Data Collection and instruments

#### 1) Data selection

Out of the total 453,888 data points provided in the raw dataset, 64,043 data points corresponding to individuals aged 19 to 35 years were selected. After excluding data with missing values in at least one of the medical examination, health survey, or nutrition survey datasets, a total of 823 data points were preprocessed and used for analysis. Of the data, 70% was randomly selected using Excel's random function and used as machine learning training data, while the remaining 30% was used as test data [14].

#### 2) Prediabetes and diabetes classification

The classification of individuals with prediabetes and diabetes was based on fasting blood glucose and HbA1c results from the medical examination data. Diabetes was defined as having a fasting blood glucose level of 126 mg/dL or higher, having received a diagnosis from a doctor, taking glucose-lowering medications, using insulin injections, or having an HbA1c level of 6.5% or higher. Prediabetes was defined as having a fasting blood glucose level between 100 and 125 mg/dL or an HbA1c level between 5.7% and 6.4%, without meeting the criteria for diabetes [13]. Since individuals with prediabetes are at a high risk of progressing to diabetes, these two conditions were combined to enhance the predictive power of the model. In this study, diabetes and prediabetes were combined into a single category referred to as “prediabetes or higher,” which was compared with the normal group. Individuals who did not meet these criteria were classified as normal.

#### 3) General characteristics

The general characteristics were analyzed using data from the health survey, including sex, age, educational level, occupation, whether the participant lived alone, marital status, and type of health insurance. Sex was used as recorded as biological sex in the raw data, while educational level, occupation, marital status, and type of health insurance were used as categorical variables based on recoded categories in the raw data. Age was used as a continuous variable.

#### 4) Dietary intake survey

The dietary intake survey utilized the 24-hour dietary recall data from the nutrition survey. Information regarding whether and why the participant followed a specific diet was used as categorical variables, as recorded in the raw data. The processed data on nutrient intake, as provided by the survey, were used directly as independent variables. The variables included daily food intake (g), daily energy intake (kcal), daily water intake (g), daily protein intake (g), daily fat intake (g), daily saturated fatty acid intake (g), daily monounsaturated fatty acid intake (g), daily polyunsaturated fatty acid intake (g), daily n-3 fatty acid intake (g), daily n-6 fatty acid intake (g), daily cholesterol intake (mg), daily carbohydrate intake (g), daily dietary fiber intake (g), daily sugar intake (g), daily calcium intake (mg), daily phosphorus intake (mg), daily sodium intake (mg), daily potassium intake (mg), daily magnesium intake (mg), daily iron intake (mg), daily zinc intake (mg), vitamin A (retinol activity equivalent) intake (μg RAE), daily vitamin D intake (μg), daily vitamin E intake (mg α-TE), daily beta-carotene intake (μg), daily retinol intake (μg), daily thiamine intake (mg), daily riboflavin intake (mg), daily niacin intake (mg), daily folate intake (μg), and daily vitamin C intake (mg).

### 4. Machine learning model

#### 1) Logistic regression

Logistic regression is used to predict the probability of a dependent variable by considering the effects of independent variables in binary classification problems. In particular, this model is useful for producing dichotomous outcomes, such as the presence or absence of a disease, and is commonly applied in various fields including healthcare and marketing. The logistic function, which forms an S-shaped curve, is used to transform the output into a probability value between 0 and 1. Based on this probability, the data is classified, and the model's performance is evaluated using metrics such as accuracy, specificity, precision, recall, and the F1 score (the harmonic mean of precision and recall). A performance value closer to 1 indicates that the model exhibits high diagnostic accuracy [14].

#### 2) k-Nearest Neighbors (kNN)

kNN is a widely used classification algorithm in supervised learning. This algorithm is instance-based, meaning it does not require the creation of a separate model but classifies or predicts new data points based on the k-nearest neighbors from the training data. It operates as a non-parametric method, using distance-based calculations such as Euclidean distance or Manhattan distance to determine proximity. kNN is more beneficial in scenarios with smaller datasets rather than large-scale data owing to its simplicity and ease of implementation [14].

#### 3) Random forest

Random forest is a widely used supervised learning algorithm for both classification and regression tasks. This model trains multiple decision trees independently and generalizes their predictions to create an optimal model, often referred to as an ensemble model. Averaging the results or using a majority vote determines the final prediction. Each tree is trained through random sampling of the data and feature selection to prevent overfitting and improve the model's generalization performance. The advantages of random forest include high prediction accuracy, stability, and strong handling of non-linear data [14].

### 5. Data analysis

This study employed the Orange 3.5 program to analyze a diabetes prediction model based on dietary intake data. Orange is an open-source data mining and machine learning tool that allows for easy data preprocessing, visualization, modeling, and evaluation. It provides an intuitive interface for efficiently managing complex data analyses [15].

**Figure 1.**
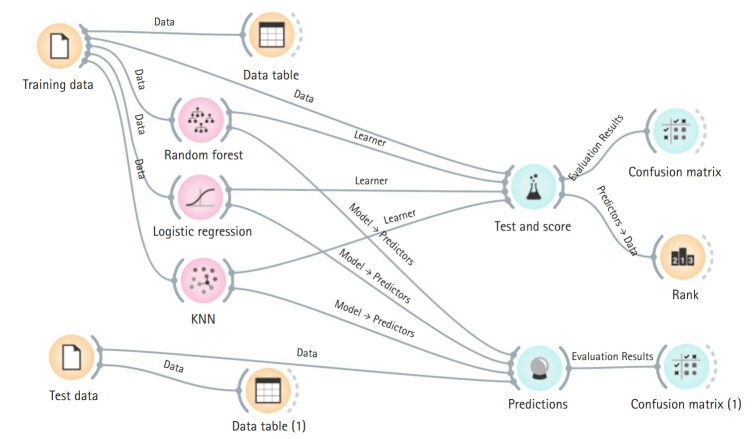
Workflow of machine learning models for prediabetes prediction using dietary intake data in the Orange 3.5 program.

To evaluate the performance of the models, a five-fold cross-validation was conducted, thereby reducing variance due to data partitioning and verifying the model's generalization capability. After cross-validation, a separate test set was used to evaluate whether the model avoided overfitting and maintained consistent performance on new data. This dual-validation process ensured the stability of the model and its practical applicability (Figure 1).

### 6. Ethical Considerations

This study is a secondary data analysis utilizing the first-year data from the 9th Korea National Health and Nutrition Examination Survey (KNHANES), publicly released by the Korea Disease Control and Prevention Agency (KDCA). The KNHANES received ethical approval from the Institutional Review Board (IRB) of the KDCA (Approval No: 2018-01-03-4C-A). De-identified data were provided to the researchers upon official authorization for research purposes, ensuring the protection of participant confidentiality. This study adhered to the ethical principles outlined in the Declaration of Helsinki. As a secondary data analysis of anonymized datasets, additional IRB approval was not required.

## RESULTS

### 1. Dataset composition and participant characteristics

#### 1) Data characteristics

In the total dataset, 14.7% were classified as prediabetic or diabetes, with 14.2% in the training dataset and 15.8% in the test dataset. Regarding sex, the total dataset comprised 45.3% male and 54.7% female, while the training dataset had 44.8% male and the test dataset had 46.6% male. For education level, 57.8% of the total dataset had a university education or higher, with 59.2% in the training dataset and 54.7% in the test dataset. In terms of occupation, 25.4% of the total dataset were professionals, and 32.2% were unemployed; the training dataset had 25.5% professionals and 34.0% unemployed, while the test dataset had 25.1% professionals and 27.9% unemployed. As for household size, 82.1% of the total dataset lived in households of two or more people, with 81.9% in the training dataset and 82.6% in the test dataset. Marital status showed 22.5% of the total dataset were married, compared to 22.4% in the training dataset and 22.7% in the test dataset (Table 1).

#### 2) Daily nutrient intake

The results of the nutrient intake data for the total dataset are as follows: water intake (cup) was 5.44 ± 3.49, food intake (g) was 1478.81 ± 723.18, energy intake (kcal) was 1899.42 ± 860.86, and water intake (g) was 1019.16 ± 585.79. Protein intake (g) was 76.03 ± 40.26, fat intake (g) was 59.88 ± 35.89, saturated fatty acid intake (g) was 19.85 ± 13.44, monounsaturated fatty acid intake (g) was 19.80 ± 13.23, and polyunsaturated fatty acid intake (g) was 14.32 ± 9.88. Intake of n-3 fatty acids (g) was 1.73 ± 1.35, n-6 fatty acids (g) was 12.51 ± 8.70, and cholesterol intake (mg) was 310.56 ± 222.60. Carbohydrate intake (g) was 242.64 ± 110.80, dietary fiber intake (g) was 18.71 ± 10.09, and sugar intake (g) was 58.09 ± 40.32. Key minerals included calcium intake (mg) at 461.68 ± 268.61, phosphorus intake (mg) at 1030.84 ± 471.83, sodium intake (mg) at 3078.38 ± 1692.97, potassium intake (mg) at 2297.17 ± 1059.54, magnesium intake (mg) at 253.65 ± 120.40, iron intake (mg) at 9.22 ± 7.65, and zinc intake (mg) at 9.72 ± 5.60.

For vitamins, vitamin A intake (μgRAE) was 373.23 ± 314.54, vitamin D intake (μg) was 2.92 ± 5.26, vitamin E intake (mg α-TE) was 7.20 ± 3.94, beta-carotene intake (μg) was 2050.29 ± 2203.79, retinol intake (μg) was 199.39 ± 250.25, thiamine intake (mg) was 1.15 ± 0.76, riboflavin intake (mg) was 1.67 ± 0.87, niacin intake (mg) was 13.43 ± 9.53, folate intake (μgDFE) was 248.47 ± 131.54, and vitamin C intake (mg) was 62.23 ± 85.94.

The training and test datasets showed similar nutrient intake patterns, as detailed in Table 2.

### 2. Prediction accuracy of algorithm models

To verify the prediction accuracy of the algorithm models using the training data, we compared the models using the performance metrics: area under the curve (AUC), classification accuracy (CA), the harmonic mean of precision and recall (F1 score), precision, and recall. The results are shown in Table 3. The logistic regression model recorded an AUC of 0.61, CA of 0.83, F1 score of 0.80, precision of 0.77, and recall of 0.83. The kNN model showed an AUC of 0.58, CA of 0.84, F1 score of 0.80, precision of 0.77, and recall of 0.84. The random forest model recorded an AUC of 0.61, CA of 0.86, F1 score of 0.80, precision of 0.85, and recall of 0.86.

**Figure 2.**
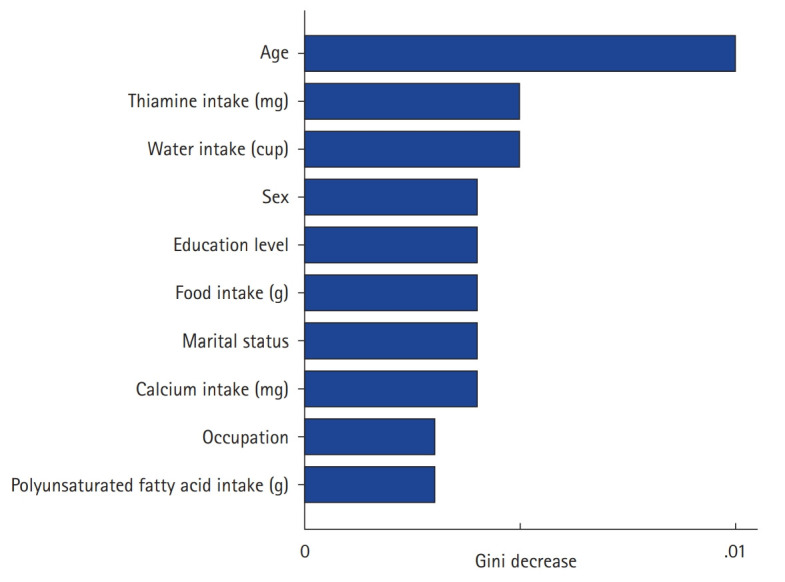
Top 10 variables by Gini decrease for predicting prediabetes in young adults.

In addition, according to the analysis of variable importance using the Gini index, age (.01), thiamine intake (mg) (.01), and water intake (cup) (.01) ranked as the top variables, and the top 10 variables are presented in Figure 2.

### 3. Validation of prediction accuracy of algorithm models

The validation results of the prediction accuracy of the algorithm models using the test data are shown in Table 3. The random forest model recorded an AUC of 0.49, CA of 0.84, F1 score of 0.77, precision of 0.71, and recall of 0.84. The logistic regression model showed an AUC of 0.54, CA of 0.82, F1 score of 0.76, precision of 0.71, and recall of 0.82. The kNN model recorded an AUC of 0.47, CA of 0.83, F1 score of 0.77, precision of 0.74, and recall of 0.83.

## DISCUSSION

Based on the key results, the following discussion is provided.

In the total, training, and test datasets, the proportion of individuals with prediabetes or higher was consistently 14%–15%. Although this study excluded missing data from individuals who did not participate in the dietary intake survey, did not undergo blood tests, or did not respond, which somewhat limits the generalizability to the entire Korean population, the Korean Diabetes Association has also reported that the prevalence of prediabetes is increasing among younger adults, highlighting the need for management as the duration of diabetes may be prolonged [3]. This indicates that various prediction methods are necessary for prevention and early intervention.

In this study, dietary intake survey data were applied to three machine learning models—logistic regression, kNN, and random forest—to predict individuals with prediabetes or higher. AUC is used to assess how well a model distinguishes between individuals with prediabetes and normal individuals, with values closer to 1 indicating better model performance. Precision represents the proportion of true positive results out of all positive predictions, while recall measures the proportion of actual positive cases that were correctly identified. These metrics help evaluate a model’s prediction accuracy and sensitivity [16,17]. Among the models, random forest achieved the highest performance, with a classification accuracy of 86%, indicating that the model can accurately distinguish between prediabetic and normal individuals. The precision of the random forest model was 85%, meaning it had a low rate of false positives, and the recall was 84%, indicating a well-balanced trade-off between precision and recall [16,17].

The logistic regression model showed a classification accuracy of .83, slightly lower than that of random forest, with a precision of .77 and a recall of .71. This suggests that logistic regression has a higher rate of false positives and may miss some individuals with prediabetes. And the kNN model demonstrated a classification accuracy of .84, with a precision of .77 and a recall of .84. While its recall is similar to that of random forest, the lower precision suggests that the kNN model may generate more false positives, potentially leading to unnecessary treatments or interventions [16,17]. These results indicate that random forest offers the best balance among the three models and is the most reliable tool for predicting prediabetes in young adults. By using this model, early intervention can be achieved, reducing unnecessary treatments and diagnostic errors.

In terms of AUC, which was employed to evaluate how well the model distinguishes between individuals with prediabetes and normal individuals [18], the logistic regression model demonstrated the highest value. An AUC value close to 1 indicates excellent classification performance, whereas a value below 0.5 suggests no classification ability [19]. As the logistic regression model showed an AUC value greater than 0.5, this suggests that this model provides more consistent performance compared with the other models. Although the logistic regression model showed somewhat lower performance in AUC and precision in the prediction validation, its overall accuracy was still acceptable. Synthesizing the results across models confirmed that the random forest model may outperform in managing the complexity of the data, while both the logistic regression and kNN models were found to maintain adequate predictive performance, suggesting their potential applicability in specific contexts.

Meanwhile, the random forest model has the advantage of handling complex interactions between various variables [20], making it suitable for managing complicated variables such as dietary intake data. However, to precisely understand the model's prediction patterns, it is necessary to refer to the variable importance determined by the Gini index [21]. Among such variables, age, followed by thiamine and water, contributed the most to predicting individuals with prediabetes or higher.

Due to the nature of machine learning analysis, it is difficult to determine the precise directionality of the variables. However, based on previous studies, it can be inferred that as individuals advance in age, insulin resistance increases and metabolic rate decreases, increasing the likelihood of progressing to prediabetes [22]. In particular, adults aged 19–35 are in a phase of life whereby lifestyle changes often occur simultaneously [23], possibly leading to increased insulin resistance and difficulty in blood sugar regulation. These factors likely explain why age emerged as an important variable in the machine learning model. Additionally, thiamine plays a crucial role in carbohydrate metabolism by acting as a coenzyme in the conversion of glucose to energy. It supports insulin secretion and helps reduce oxidative stress and inflammation, which are exacerbated by hyperglycemia and contribute to diabetic complications. Research has shown that thiamine enhances hexokinase activity, increases insulin secretion, and restores normal enzyme function in the liver and kidneys of diabetic models, thereby alleviating pathological abnormalities associated with diabetes [24]. Therefore, a deficiency in thiamine may result in impaired insulin secretion, leading to problems in glucose metabolism, and potentially accelerating the progression to prediabetes. This likely explains why thiamine intake emerged as an important variable in this study. Similarly, adequate water intake is known to help in maintaining stable blood sugar levels by diluting glucose concentrations in the bloodstream [25], which may account for its significance as a key variable in the machine learning model.

By considering the connection between physiological mechanisms and individual characteristics, as well as dietary intake patterns, blood sugar regulation can be approached from a physiological perspective based on the consumption of specific nutrients. Although the contribution scores of carbohydrates and sugar intake were low in this study, these nutrients rapidly increase blood glucose levels, as previous studies have shown. However, as various nutrients such as fats and dietary fibers interact in a complex manner to influence blood glucose regulation [26], by utilizing the random forest machine learning model, which employs an ensemble learning method with multiple decision trees to learn dietary patterns, it is expected to effectively reflect multidimensional interactions and provide more accurate predictions of prediabetes. This study highlights the utility of machine learning models for predicting prediabetes in young adults using dietary intake data, which are relatively easy to collect. According to previous studies, while older adults may develop prediabetes due to age-related metabolic changes, younger adults are primarily influenced by lifestyle factors such as diet and physical activity [22,23]. The results of this study emphasize the importance of addressing these factors early in life to prevent the progression to diabetes. This finding has significant implications for public health policy development, as it enables early intervention for the prevention of chronic diseases among young adults, a group often overlooked in healthcare. Key variables identified in this study, such as age, thiamine, and water, play a critical role in predicting prediabetes and should be incorporated into future predictive models. By properly utilizing these variables, the predictive performance of the models can be further enhanced, allowing for more accurate predictions of prediabetes and thereby strengthening early intervention and prevention strategies.

Overall, this study contributes to the theoretical development of disease prevention and health management strategies by validating a model for predicting prediabetes. It also provides a foundation for future research by analyzing multidimensional variable interactions, offering new insights into diabetes prediction in young adults. Practically, this study offers foundational data that nurses can use as a tool for early intervention and personalized health education, helping to improve patient health outcomes effectively.

However, the relatively lower performance of the random forest model in terms of AUC and precision indicates that caution is needed when considering its clinical application. Future research should aim to enhance the model's reliability and conduct further validation. While the 24-hour dietary recall method provides detailed nutritional data, it has the limitation of relying on participants' memory, which may reduce accuracy [27]. Therefore, future studies could improve the model by incorporating additional data, including psychological factors such as stress and depression, as well as physical activity and lifestyle factors that influence diabetes risk. Moreover, this study focused exclusively on young adults aged 19 to 35, limiting the generalizability of the findings to other age groups. As such, care must be taken when applying the results to the prediction of prediabetes across different age ranges.

## CONCLUSION

In this study, we compared machine learning models to predict prediabetes in young adults in Korea using dietary intake data from the KNHANES. Among the models evaluated, the random forest model demonstrated the highest predictive accuracy and precision compared with logistic regression and kNN models. The key predictive variables were age, thiamine intake, and water intake, which contributed significantly to the model's performance. Based on these results, we confirmed the feasibility of predicting prediabetes in young adults and provided foundational data for early intervention in diabetes prevention. However, given the need for improvement in certain performance metrics, such as AUC, we recommend that future studies incorporate additional variables, such as physical activity, lifestyle factors, and psychological variables, to enhance the predictive power of the models.

## Figures and Tables

**Table 1.** General Characteristics of Participants (N = 823)

Variables	Type	Role	Categories	Total (n = 823)	Training data (n = 576)	Test data (n = 247)
Diabetes	Categorical	Target	Prediabetes or diabetes	121 (14.7)	82 (14.2)	39 (15.8)
			Normal	702 (85.3)	494 (85.8)	208 (84.2)
Sex	Categorical	Feature	Men	373 (45.3)	258 (44.8)	115 (46.6)
			Women	450 (54.7)	318 (55.2)	132 (53.4)
Education level	Categorical	Feature	Elementary and below	3 (0.4)	2 (0.3)	1 (0.4)
			Middle school	8 (1.0)	4 (0.7)	4 (1.6)
			High school	336 (40.8)	229 (39.8)	107 (43.3)
			College or higher	476 (57.8)	341 (59.2)	135 (54.7)
Occupation	Categorical	Feature	Manager, professional and related workers	209 (25.4)	147 (25.5)	62 (25.1)
			Office workers	134 (16.3)	97 (16.8)	37 (15.0)
			Service and sales workers	127 (15.4)	73 (12.7)	54 (21.9)
			Skilled workers, machinery operators, and assemblers	53 (6.4)	41 (7.1)	12 (4.9)
			Simple labor workers	35 (4.3)	22 (3.8)	13 (5.3)
			Unemployed (housewives, students, etc.)	265 (32.2)	196 (34.0)	69 (27.9)
Single household	Categorical	Feature	Single-person household	147 (17.9)	104 (18.1)	43 (17.4)
			Two or more people	676 (82.1)	472 (81.9)	204 (82.6)
Marital status	Categorical	Feature	Married	185 (22.5)	129 (22.4)	56 (22.7)
			Single	638 (77.5)	447 (77.6)	191 (77.3)
Health insurance	Categorical	Feature	National health insurance (local)	213 (25.9)	158 (27.4)	55 (22.3)
			National health insurance (workplace)	581 (70.6)	397 (68.9)	184 (74.5)
			Medical aid	29 (3.5)	21 (3.7)	8 (3.2)
Dietary therapy	Categorical	Feature	Yes	248 (30.1)	165 (28.7)	83 (33.6)
			No	575 (69.9)	411 (71.4)	164 (66.4)
Reason for dietary therapy	Categorical	Feature	Disease	17 (2.1)	11 (1.9)	6 (2.4)
			Weight control	219 (26.6)	146 (25.4)	73 (29.6)
			Others	12 (1.5)	8 (1.4)	4 (1.6)
			Not applicable	575 (69.9)	411 (71.3)	164 (66.4)
Age	Numeric	Feature		27.20 ± 4.78	27.21 ± 4.68	27.19 ± 5.02

Values are presented as the mean ± standard deviation or n (%). Percentages may not sum to 100% due to rounding.

**Table 2.** Dietary Intake Characteristics of Participants (N = 823)

Variable (/1 day)	Total (n = 823)			Training data (n = 576)			Test data (n = 247)		
(Type = numeric, Role = feature)	Min	Max	M ± SD	Min	Max	M ± SD	Min	Max	M ± SD
Water intake (cup)	0.00	30.00	5.44 ± 3.49	0.50	30.00	5.47 ± 3.46	0.00	30.00	5.37 ± 3.58
Food intake (g)	183.70	6895.63	1478.81 ± 723.18	183.70	4806.91	1464.52 ± 698.80	273.52	6895.63	1512.12 ± 777.52
Energy intake (kcal)	264.80	6757.00	1899.42 ± 860.86	264.80	5637.90	1882.74 ± 839.54	528.85	6757.00	1938.33 ± 909.20
Water intake (g)	25.60	5666.10	1019.16 ± 585.79	25.60	3798.33	1006.58 ± 558.53	65.47	5666.09	1048.49 ± 645.15
Protein intake (g)	7.03	299.54	76.03 ± 40.26	7.03	299.54	74.95 ± 40.17	21.31	253.18	78.56 ± 40.44
Fat intake (g)	7.71	248.96	59.88 ± 35.89	7.71	248.96	59.71 ± 35.43	10.43	223.59	60.29 ± 37.02
Saturated fatty acid intake (g)	2.14	100.48	19.85 ± 13.44	2.14	100.48	20.00 ± 13.60	2.42	75.30	19.50 ± 13.06
Monounsaturated fatty acid intake (g)	1.76	93.70	19.80 ± 13.23	1.76	87.13	19.88 ± 13.12	2.50	93.70	19.61 ± 13.50
Polyunsaturated fatty acid intake (g)	0.25	90.97	14.32 ± 9.88	0.25	60.02	13.93 ± 8.88	1.81	90.97	15.21 ± 11.85
N-3 fatty acid intake (g)	0.04	12.50	1.73 ± 1.35	0.04	8.27	1.65 ± 1.20	0.06	12.50	1.91 ± 1.63
N-6 fatty acid intake (g)	0.21	80.89	12.51 ± 8.70	0.21	52.38	12.21 ± 7.86	1.67	80.89	13.22 ± 10.39
Cholesterol intake (mg)	0.00	2447.99	310.56 ± 222.60	0.00	1392.19	300.45 ± 209.05	0.00	2447.99	334.13 ± 250.26
Carbohydrate intake (g)	30.03	883.33	242.64 ± 110.80	30.03	883.33	240.40 ± 108.04	54.53	797.07	247.87 ± 117.06
Dietary fiber intake (g)	0.99	68.56	18.71 ± 10.09	0.99	68.56	18.60 ± 10.02	3.34	57.76	18.98 ± 10.24
Sugar intake (g)	3.63	328.78	58.09 ± 40.32	4.53	328.78	58.05 ± 41.30	3.63	198.37	58.18 ± 38.01
Calcium intake (mg)	62.64	2359.11	461.68 ± 268.61	62.87	2359.11	456.09 ± 271.46	62.64	1326.26	474.72 ± 261.94
Phosphorus intake (mg)	140.76	3467.33	1030.84 ± 471.83	140.76	3467.33	1015.69 ± 467.91	267.95	3435.25	1066.16 ± 479.94
Sodium intake (mg)	134.13	9702.56	3078.38 ± 1692.97	134.13	9496.70	3060.87 ± 1673.57	238.33	9702.56	3119.22 ± 1740.14
Potassium intake (mg)	267.26	8276.79	2297.17 ± 1059.54	267.26	8276.79	2284.03 ± 1056.01	559.84	6190.34	2327.80 ± 1069.24
Magnesium intake (mg)	23.22	787.94	253.65 ± 120.40	23.22	787.94	249.56 ± 116.92	53.57	780.26	263.17 ± 127.88
Iron intake (mg)	0.22	108.05	9.22 ± 7.65	0.22	108.05	9.24 ± 8.48	1.91	26.71	9.16 ± 5.23
Zinc intake (mg)	0.75	46.43	9.72 ± 5.60	0.75	46.43	9.72 ± 5.64	1.68	35.01	9.71 ± 5.53
Vitamin A (retinol activity equivalents) intake (μgRAE)	14.09	2969.08	373.23 ± 314.54	14.09	2298.14	354.15 ± 276.23	21.12	2969.08	417.73 ± 386.51
Vitamin D intake (μg)	0.00	68.98	2.92 ± 5.26	0.00	68.98	2.83 ± 5.29	0.00	60.48	3.14 ± 5.20
Vitamin E intake (mg α-TE)	0.76	32.90	7.20 ± 3.94	0.76	24.38	7.12 ± 3.72	1.94	32.90	7.38 ± 4.40
Beta-carotene intake (μg)	0.71	25666.85	2050.29 ± 2203.79	0.71	15906.43	1913.97 ± 1942.03	8.00	25666.85	2368.17 ± 2695.93
Retinol intake (μg)	0.00	2722.48	199.39 ± 250.25	0.00	2113.19	191.70 ± 220.62	0.00	2722.48	217.33 ± 308.26
Thiamine intake (mg)	0.12	7.79	1.15 ± 0.76	0.12	7.79	1.17 ± 0.78	0.20	5.83	1.10 ± 0.71
Riboflavin intake (mg)	0.26	6.53	1.67 ± 0.87	0.26	5.96	1.65 ± 0.84	0.27	6.53	1.70 ± 0.95
Niacin intake (mg)	0.04	124.44	13.43 ± 9.53	0.04	69.10	13.17 ± 8.51	1.55	124.44	14.06 ± 11.58
Folate intake (μgDFE)	19.89	1148.79	248.47 ± 131.54	19.89	814.66	246.97 ± 128.68	36.60	1148.79	251.97 ± 138.18
Vitamin C intake (mg)	0.00	901.67	62.23 ± 85.94	0.00	613.48	59.53 ± 76.16	0.13	901.67	68.54 ± 105.18

Min = Minimum; Max = Maximum; M = Mean; SD = Standard deviation; g = Grams; kcal = Kilocalories; μgRAE = Micrograms retinol activity equivalent; α-TE = Alpha-tocopherol equivalents; μgDFE = Micrograms dietary folate equivalent.

**Table 3.** Prediction Accuracy Validation for the Training and Test Datasets

Dataset	Model	AUC	CA	F1	Precision	Recall
Training data	Logistic regression	.61	.83	.80	.77	.83
	kNN	.58	.84	.80	.77	.84
	Random forest	.61	.86	.80	.85	.86
Test data	Random forest	.49	.84	.77	.71	.84
	Logistic regression	.54	.82	.76	.71	.82
	kNN	.47	.83	.77	.74	.83

AUC = Area under the curve; CA = Classification accuracy; F1 = Harmonic mean of precision and recall; kNN = k-Nearest Neighbors.

## Data Availability

The data that support the findings of this study are available from the corresponding author upon reasonable request.
